# Body composition measurements and risk of hematological malignancies: A population-based cohort study during 20 years of follow-up

**DOI:** 10.1371/journal.pone.0202651

**Published:** 2018-08-23

**Authors:** Hannes Hagström, Anna Andreasson, Axel C. Carlsson, Mats Jerkeman, Mattias Carlsten

**Affiliations:** 1 Department of Medicine, Solna, Karolinska Institutet, Stockholm, Sweden; 2 Stress Research Institute, Stockholm University, Stockholm, Sweden; 3 Department of Psychology, Macquarie University, North Ryde, NSW, Australia; 4 Division of Family Medicine and Primary Care, Department of Neurobiology, Care Sciences and Society (NVS), Karolinska Institutet, Stockholm, Sweden; 5 Department of Medical Sciences, Cardiovascular Epidemiology, Uppsala University, Uppsala, Sweden; 6 Department of Oncology, Clinical Sciences, Lund University, Lund, Sweden; 7 Center for Hematology and Regenerative Medicine, Department of Medicine, Huddinge, Karolinska Institutet, Stockholm, Sweden; Temple University School of Medicine, UNITED STATES

## Abstract

High body mass index (BMI) is associated with development of hematological malignancies (HMs). However, although BMI is a well-established measurement of excess weight, it does not fully reflect body composition and can sometimes misclassify individuals. This study aimed at investigating what body composition measurements had highest association with development of HM. Body composition measurements on 27,557 individuals recorded by healthcare professionals as part of the Malmö Diet and Cancer study conducted in Sweden between 1991–1996 were matched with data from national registers on cancer incidence and causes of death. Cox regression models adjusted for age and sex were used to test the association between one standard deviation increments in body composition measurements and risk of HM. During a median follow-up of 20 years, 564 persons developed an HM. Several body composition measurements were associated with risk of developing an HM, but the strongest association was found for multiple myeloma (MM). Waist circumference (HR 1.31, p = 0.04) and waist-hip ratio (HR 1.61, p = 0.05) had higher risk estimates than BMI (HR 1.18, p = 0.07) for MM. In conclusion, our study shows that measurements of abdominal adiposity better predict the risk of developing HM, particularly MM, compared to BMI.

## Introduction

Hematological malignancy (HM) is a common cause of morbidity and mortality, and includes neoplasia of both myeloid and lymphoid origin such as leukemia, lymphoma and plasma cell diseases. A slight incline in the incidence of several HMs, including non-Hodgkin lymphoma (NHL) and multiple myeloma (MM), has been noted during the last decades[1]. There is limited evidence for why people develop HM, however age, male sex, ethnicity, family history of cancer including HM, autoimmune diseases, exposure to petrol products, radiation or alkylating chemotherapy as well as treatment with immunosuppression have been recognized as potential risk factors. Hence, known modifiable risk factors are scarce. However, recently, and in congruence with reports on cancer in general[2–4], overweight and obesity have been implicated as risk factors for development of several HMs, such as lymphoma[3, 5–7], leukemia, including acute promyelocytic leukemia[8, 9] and MM[3].

Excess body weight is popularly recorded as body mass index (BMI). Most of the previous evidence on the association between a high BMI and risk of HM comes from self-reported data on weight, height or BMI, which is known to be prone to recollection and misclassification biases[10, 11]. Also, BMI as a marker for obesity has been criticized, as it may misclassify persons with a high muscular mass as overweight and in some cases even obese[10, 11]. Abdominal obesity is well-known to be a better risk factor than BMI for development of cardiovascular[12] and liver disease[13] as well as for dementia[14]. Hence, we hypothesized that abdominal adiposity would better predict risk for HM compared to BMI.

The aim of this study was to investigate what body composition measurements that best predict development of HM, using a population-based cohort study where body measurements were objectively documented by health care professional and not self-reported by the study subjects. We also aimed at addressing the relationship between these measurements and specific HMs such as leukemia, lymphoma and MM.

## Material and methods

### Participants and procedure

The present study is based on data from the EPIC cohort study[15], part of the Malmö Diet and Cancer study, which invited all adult inhabitants of the city of Malmö, Sweden in predefined age ranges. The cohort has previously been described in detail[16–18]. Briefly, during March 1991 and September 1996, 28,098 individuals (women: n = 17,035, born 1923–1950; men: n = 11,063, born 1923–1945) attended a baseline examination, underwent measurements of body composition, and filled out a self-administered questionnaire. Trained nurses at a screening center performed the examinations.

All Swedish citizens are assigned a personal identity number at birth[19]. This allows for linkage between study cohorts and population-based national registers. The Cancer Register (CR) was established in 1958, and includes information on all diagnosed malignancies classified according to International Classification of Diseases (ICD) codes, 7–10. The Causes of Death Register (CDR) contains data from 1961 onwards regarding the causes of death of all Swedish citizens, including if the person died abroad. It is mandatory for the responsible physician to report the underlying cause of death (e.g. ischemic heart disease) and any disease that could have contributed to the death of the individual (e.g. hypertension). Follow-up data was acquired from these national registries until December 31^th^, 2014. The study followed the ethical guidelines in the Helsinki declaration. The ethics committee at Lund University approved the study (LU 51/90), and all participants provided informed consent.

### Variables

#### Body composition measurements

All body composition measurements were objectively sampled at baseline by trained study nurses. Standing height was measured with a fixed stadiometer calibrated in centimeters (cm). Weight was measured to the nearest 0.1 kg using balance-beam scales with participants wearing light clothing and no shoes. *BMI* was calculated as weight (kg) divided by the square of the height (m^2^). Waist circumference (WC) was defined as the circumference (cm) between the lowest rib margin and iliac crest, and hip circumference (cm) as the largest circumference between waist and thighs. Waist-hip ratio (WHR) was defined as the ratio of circumference of waist to hip. Waist-to-height ratio (WHtR) was defined as the WC divided by height, and waist-to-hip-to-height ratio (WHHR) was defined as the WHR divided by height. The A Body Shape Index (ABSI) was defined as WC/((BMI^(2/3)^ x height^(1/2)^)[20]. Bioelectrical impedance analyzers (BIA) were used to estimate body composition and body fat per cent (BFP) was calculated using an algorithm according to procedures provided by the manufacturer (BIA 103, single-frequency analyzer, JRL Systems, USA)[21].

#### Covariates

Data on smoking was retrieved from the self-administered questionnaire at baseline. Smoking status was defined as being a current smoker, former smoker or having never smoked. Alcohol consumption was assessed using a validated questionnaire asking about alcohol consumption during the last 30 days[22] and was quantified in grams per day. Higher education was defined as secondary or further secondary education (approximately 11 years or more). Leisure time physical activity score was assessed as number of minutes of 18 different activities multiplied with an activity coefficient. The score was analyzed as low (quintile 1), moderate (quintile 2–4) or high (quintile 5) as previously described[23].

#### Hematologic malignancies

Cases of HM were ascertained from the CR and the CDR, and divided into the following subcategories and corresponding ICD 10 codes including subclasses: MM [C90.0], myeloid malignancies (including myeloid leukemia [C92], and myelodysplastic syndrome [D46.6, D46.7, D46.9, C93.1]) and lymphoid malignancies (including Hodgkin and non-Hodgkin lymphomas [C81-83, C85, C88] and lymphatic leukemia [C91]), or the corresponding ICD 7–9 codes (S1 Table). For subsequent subgroup analyses, cases with an HM diagnosis that did not fall into the categories of myeloid malignancies, NHL or MM, were excluded due to low power. However, these cases were all included in the main analysis of all HMs.

#### Statistical analysis

Cox regression models were used to test the different anthropometric measurements as predictors of all HMs and separately for MM, myeloid and lymphoid malignancies. One crude and one adjusted model were calculated per anthropometric measurements. Covariates in the multivariate analysis were selected a priori, and included age and sex. Also, we evaluated a number of other covariates for inclusion in the multivariate model including alcohol consumption, smoking status, higher education and physical activity but as neither of these changed the estimates by more than 10%, giving no indication of confounding, these were not included in further analysis. In addition, a multivariate cubic regression spline model adjusted for age and sex with three degrees of freedom and an alpha-level of 0.05 was calculated per measure to test for linearity.

Harrell’s C-statistics[24] were used to find the strongest predictor of future HM. Likelihood ratio tests were used to evaluate if the models including each anthropometric measurements predicted development of HMs better than a model using only age and sex. To better compare the different body composition measurements, they are all presented as standardized measures and thus hazard ratios reflect changes per standard deviation. HRs for non-standardized values of BMI and WC are also presented to increase readability. In addition, HRs for BMI, WC and WHR are presented per categories for comparison. For BMI, results were categorized as below 18.5 kg/m^2^, between 18.5–25 kg/m^2^, between 25–30 kg/m^2^ and above 30 kg/m^2^. For WC results were categorized as above or below the defined cut-off stratified on sex (>94 cm for men and >80 cm for women). The cut-off for WHR was defined as 0.90 for men and 0.80 for women.

Time of follow-up began at recruitment and ended at the time of first diagnosis of HM, death, emigration or end of follow-up (31 December, 2014). Persons that died during the follow-up from other causes than HM were censored. Emigrants were considered lost to follow-up but contributed with follow-up time until the date of emigration. As a sensitivity analysis, we excluded all cases with a diagnosis of any HM within one year after baseline.

## Results

### Study cohort

At baseline, there were 28,098 persons of whom 481 persons were excluded from the analyses because of missing data on body composition measurements or covariates. Of the 27,617 persons with complete data, 60 cases had been diagnosed with an HM prior to baseline and were therefore excluded. In the final cohort of 27,557 individuals, there were 16,750 women (60.8%) and 10,807 men (39.2%). Mean age was 58.1 years and mean BMI was 25.7 kg/m^2^. The majority (88%) were born in Sweden and about one third (28.2%) were current smokers. Almost half (46.7%) of women and 62.4% of men were overweight as defined by BMI ≥25 kg/m^2^. Baseline data for the entire cohort stratified on sex (n = 27,557) is presented in Table 1.

**Table 1:** Descriptive statistics at baseline in 27,557 Swedish men and women.

	Women (n = 16750)		Men (n = 10807)		
	Mean / n	SD / %	Mean / n	SD / %	P^a^
**Age (years)**	57.4	7.9	59.2	7.1	<0.001
**Body composition measurements**					
**BMI (kg/m**^**2**^**)**	25.4	4.2	26.4	3.5	<0.001
**Waist (cm)**	77.8	10.5	93.7	10	<0.001
**Hip (cm)**	97.9	9.6	99.3	7.1	<0.001
**WHR (%)**	0.79	0.1	0.94	0.1	<0.001
**WHtR (cm/m)**	0.48	0.07	0.53	0.07	<0.001
**WHHR (%)**	0.49	0.04	0.53	0.04	<0.001
**ABSI (value)**	0.071	0.004	0.08	0.004	<0.001
**Body fat per cent (%)**	30.1	5	20.7	5	<0.001
**Covariates**					
**Alcohol (gram/day)**	7.7	8.7	15.6	16.1	<0.001
**Smoking (current)**	4692	28	3083	28.5	<0.001
**Smoking (past)**	4641	27.7	4680	43.3	
**Smoking (never)**	7417	44.3	3044	28.2	
**Higher education (yes)**	5101	30.5	3752	34.7	<0.001
**Physical activity**					
**low**	3287	19.6	2236	20.7	<0.001
**medium**	10328	61.7	6200	57.4	
**high**	3135	18.7	2371	21.9	

^a^Differences between continuous parameters were investigated using the Mann-Whitney U-test. Differences between categorical variables were examined using the Chi2 test. Abbreviations: BMI, body mass index. WHR, waist-hip ratio. WHHR, waist-to-hip-to-height ratio. WHtR, Waist-to-height ratio. ABSI, a body shape index. BFP, body fat per cent. HR, hazard ratios. CI, confidence interval. LR-test, likelihood ratio test.

The cohort was followed for a median time of 19.8 years (interquartile range 18.2–21.4, range 0–23.8), corresponding to 507,063 person-years. In total, 7,968 persons (29.2%) died and 249 persons (0.9%) emigrated. In all, 564 persons (2.1%) developed an HM, including 300 women (1.8%) and 264 men (2.4%, p<0.001 for difference between men to women), and 11 persons developed two HMs. Of these in total 575 cases there were 107 cases of MM, 129 cases of myeloid malignancies (including AML, MDS, CML) and 299 cases of non-Hodgkin lymphoma (NHL) (Hodgkin and T cell lymphoma were excluded) for subtype analysis (S1 Table and S1 Fig). In addition, there were 8 cases with acute lymphatic leukemia, 13 cases with Hodgkin lymphoma, 1 case with T-cell lymphoma and 7 cases with unspecified hematologic malignancies. These 29 cases were all included in the main analysis of all HMs but were exluded in the subclassification analysis as they did not fit into any predefined category and their number was to low. ICD codes used to define outcomes are listed in S2 Table. At the end of the follow-up period, 210 of 564 (37.2%) persons that had developed an HM were alive. A similar proportion of the population included in the analyses developed an HM during follow-up compared to those excluded due to missing data (2.0% among individuals with missing data vs 2.1% among individuals with complete data).

### Body composition variables and risk of developing any hematological malignancy

In multivariate analysis, the variables that best predicted development of any HM were WC and ABSI, both based on size of hazard ratio and on C-statistics (aHR for WC 1.15 per increment standard deviation, 95% CI 1.03–1.29, p = 0.01, C-statistic 0.6221 and aHR for ABSI 1.19 per standard deviation, 95% CI 1.01–1.42, p = 0.04, C-statistic 0.6225). None of the other measurements evaluated in this study, including BMI, WHR, WHHR, WHtR, and BFP, showed significant associations with risk of developing HM. The results are presented in Table 2. No evidence against linearity were found for any of the associations in the multivariate regression spline model (p>0.05 for all measures).

**Table 2:** Hazard ratios for development of any hematological malignancy in 27,557 Swedish men and women followed for a median time of 19.8 years.

	HR	95%CI	HR, p-value	LR-test, p-value
**Null**				
**BMI**	1.06	0,98–1,16	0.15	0.16
**WC**	1.15	1.03–1.29	0.01	0.02
**WHR**	1.19	0.96–1.47	0.11	0.11
**WHHR**	1.04	0.88–1.24	0.63	0.63
**WHtR**	1.1	1.00–1.22	0.06	0.06
**ABSI**	1.19	1.01–1.42	0.04	0.04
**BFP**	1.02	0.90–1.15	0.77	0.77

Estimates adjusted for age and sex. Null model included only age and sex. HRs represent hazard ratios for an one increment increase in standard deviation per anthropometric measure. LR-test for significance of adding respective measure to the null model. Abbreviations: BMI, body mass index. WC, waist circumference. WHR, waist-hip ratio. WHHR, waist-to-hip-to-height ratio. WHtR, Waist-to-height ratio. ABSI, a body shape index. BFP, body fat per cent. HR, hazard ratios. CI, confidence interval. LR-test, likelihood ratio test.

### Waist measurements are associated with increased risk for MM, but not for myeloid malignancies or non-Hodgkin lymphoma

Next, we conducted a sub-analysis of all individuals with HM that could be grouped into myeloid malignancies, NHL or MM. This analysis revealed that the risk of developing MM was associated with waist measurements (WC, WHR and WHtR) (Table 3), while no other body composition measurement, including BMI, was associated with development of myeloid malignancies or NHL (S3A and S3B Table).

**Table 3:** Hazard ratios for development of multiple myeloma in 27,557 Swedish men and women followed for a median time of 19.8 years.

	HR	95%CI	HR, p-value	LR-test, p-value
**Null**				
**BMI**	1.18	0.99–1.42	0.07	0.08
**WC**	1.31	1.02–1.68	0.04	0.04
**WHR**	1.61	1.00–2.58	0.05	0.05
**WHHR**	1.37	0.93–2.00	0.11	0.11
**WHtR**	1.26	1.00–1.58	0.05	0.05
**ABSI**	1.24	0.84–1.83	0.27	0.28
**BFP**	1.01	0.77–1.33	0.95	0.95

Estimates adjusted for age and sex. Null model included only age and sex. HRs represent hazard ratios for an one increment increase in standard deviation per anthropometric measure. LR-test for significance of adding respective measure to the null model. Abbreviations: BMI, body mass index. WC, waist circumference. WHR, waist-hip ratio. WHHR, waist-to-hip-to-height ratio. WHtR, Waist-to-height ratio. ABSI, a body shape index. BFP, body fat per cent. HR, hazard ratios. CI, confidence interval. LR-test, likelihood ratio test.

In contrast to several previous reports, we did not observe a significantly increased risk of developing MM among individuals with BMI ≥25 kg/m^2^ (aHR 1.18, 95% CI 0.99–1.42, p = 0.07, C-statistic 0.6452). However, when further dividing BMI into groups of those with BMI <18.5, 25–29.9 and >30 kg/m^2^ and compared that to the reference group with BMI of 18.5–24.9 kg/m^2^ we identified that individuals with BMI >30 kg/m^2^ had a statistically significant risk of developing MM (aHR 1.94, 95% CI 1.17–3.32, p = 0.01) while persons with a BMI between 25–30 did not (S4 Table). However, importantly, in the subgroup of persons with a BMI between 25 to 30 kg/m^2^ (n = 10,917), standardized WHR was significantly associated with MM (aHR 2.25, 95% CI 1.01–5.01, p = 0.047) while standardized BMI was not (aHR 0.97, 95% CI 0.40–2.36, p = 0.95).

### Sensitivity analyses

Excluding cases who received a diagnosis of any HM up to one year after baseline (n = 27) did not yield any significant differences in the estimates (Data not shown).

## Discussion

High BMI has been linked to increased risk of cancer, including for most HMs[4, 25]. However, although BMI is a well-established estimate of overweight and obesity, BMI does not fully reflect the body composition of an individual and can in some cases even be incorrect[10, 11]. Furthermore, most studies on the relationship between high BMI and risk of HM are based on self-reported BMI data, an approach that have been associated with misclassification bias[10, 11]. Here we have investigated alternative measurements of excess body fat with the aim of more precisely link obesity to risk of HM. By using measurements of body composition registered by educated health care professionals we avoided the plausible bias inherent to self-reported data. In our cohort of 27,556 individuals followed for 20 years, we identify measurements taking waist circumference into consideration as better determinants than BMI for the risk of developing HM in general and MM in particular. Collectively, our report suggests a role for abdominal adiposity in predicting the risk of development of an HM, in particular MM.

A plethora of published studies support a relationship between elevated BMI and risk of developing HMs[25]. However, only a few studies have addressed the role for other measurements that potentially better reflect the type of excess body fat an individual has. One study showed that pathological WC was associated with increased risk for myeloid leukemia, whereas no association was observed with lymphoid malignancies[26]. The lack of association between waist measurements and risk of NHL has been confirmed in other studies[27, 28]. In contrast, data from the literature indicate an association between WC and risk of MM[29, 30]. Waist-related measurements, such as WC, WHR and WHtR, are more reflective of abdominal obesity as compared to BMI[31, 32]. Although a high BMI often identify individuals with abdominal adiposity, this measure is not always optimal as exemplified by BMIs among younger well-trained non-adipose adults that often can be 25 or higher. Such misclassification may flaw studies on relationships between excess body fat and risk of disease. Moreover, as could be appreciated from the literature, although overweight and obesity has been linked to risk of HM, most of these studies report the strongest, and sometime only, correlation to risk of disease among individuals with BMI ≥30 kg/m[25]. As observed in our study for MM, several studies have reported dose-response relations between BMI and risk for HM where individuals with BMI ≥30 kg/m^2^ have higher risk than those with BMI of 25–29.9 kg/m^2^ [25]. This may reflect a factual relationship, but could also be influenced or biased by non-adipose individuals with a slightly elevated BMI (e.g. BMI of 25–29.9 kg/m^2^).

The mechanism behind excess body weight and increased risk of cancer, including HMs, remains to be elucidated. Studies have linked increased glucose levels to higher risk of developing lymphoma and MM[33, 34] and a potential role for adiponectin in the development of several HMs, including MM, has been discussed[35, 36]. Moreover, high levels of leptin, a cytokine-like anorexigenic hormone released by adipocytes, have been associated with an increased risk of NHL and MM[37] and experimental studies have shown this hormone directly promotes growth of leukemic cells[38]. Other mechanisms, such as dietary pattern that has been shown to influence the risk of Hodgkin lymphoma, cannot be excluded[39]. Although not observed in our study, reports also support a positive effect of physical activity in reducing the risk of HM[40]. Identifying biological mechanisms involved and how they can be controlled or prevented is critical to potentially reduce the incidence of HMs.

Our study has several advantages over most studies published so far as body composition measurements in this study of 27,557 individuals followed for 20 years were recorded by health care professionals using standardized methods and calibrated instruments. Also, our study sample came from a population-based cohort, reducing selection bias. Moreover, as there are validated, population-based registers of high quality in Sweden and low rate of emigration, we were able to follow-up the absolute majority of individuals enrolled. Furthermore, the good health status of the Swedish population reduces the rate of potential risk factors that could bias our results. Hence, our cohort was homogenous with relatively few confounders and complete with only a few missing data points at the end of study. Nevertheless, it should be noted that this study, like for many other studies that assess body weight and risk of developing HM, is relatively small which potentially limits the power when analyzing HM subtypes. Despite this, we found an association between body composition measurements and risk of developing MM, while this was not found for the larger cohort of NHL. The latter may indicate that body composition measurements are stronger predictors for MM or that they are not relevant for the risk of developing NHL, but might also reflect that NHL is a heterogenous group of malignancies with different risk factors and that anthtropometric measurements might be related to some but not all NHLs.

Changes in the respective body composition measurements may have occurred over the study period, which we did not have data on. These factors limited our ability to in detail investigate the association between body composition measurements and risk of developing HM. What time point in life and the duration an individual need to have excess body fat also remain unclear. Data from Maskarinec et al., where body weight at 21 years of age was shown to better predict the risk of developing NHL compared to weight at inclusion of a prospective study later in life[41], indicate that early excess body weight rather than weight gain later in life impose a significant risk for developing NHL. A similar finding was reported in a cohort where risk for NHL was linked to BMI at age 20[42]. On the contrary, another study showed that BMI at 20 and 50 years of age as well as at entry of the study, all predicted increased risk for the development of NHL[43]. This may indicate that most people that are overweight or obese in early adulthood for most part stay overweight or obese also later in life. An alternative approach would therefore be to study how temporary or sustained weight loss among individuals with excess body fat impacts on the risk of developing an HM. Only a few studies have addressed this and show that sustained weight loss among obese triggered by bariatric surgery or exercise combined with reduced caloric intake reduce the risk of developing cancer[44, 45]. This may be indicative of that weight loss even later in life can reduce the risk of cancer.

## Conclusion

Based on our data, we propose that measurements that take abdominal adiposity into account better predict the risk of developing an hematological malignancy, especially multiple myeloma compared to BMI.

## Supporting information

S1 Fig Flow-chart on enrollment to the study. Flow-chart on the total number of cases enrolled on the study and the number of, and reasons for, excluding enrolled cases from final analyses.
(PDF)

S1 Table Total number of cases with hematological malignancy and the number per subgroup of hematological malignancy. (DOCX)

S2 Table ICD codes used to define outcomes. (DOCX)

S3 Table (a) Hazard ratios for development of any lymphoid malignancy in 27,557 Swedish men and women followed for a median time of 19.8 years. (b) Hazard ratios for development of any myeloid malignancy in 27,557 Swedish men and women followed for a median time of 19.8 years.
(DOCX)

S4 Table Associations between BMI, WHR and WC and development of multiple myeloma using non-standardized units. (DOCX)
