# Supplementary figures and images for: Body composition measurements and risk of hematological malignancies: A population-based cohort study during 20 years of follow-up
Source: PLoS One. 2018 Aug 23;13(8):e0202651. doi: 10.1371/journal.pone.0202651 (PMC6107196; doi:10.1371/journal.pone.0202651)

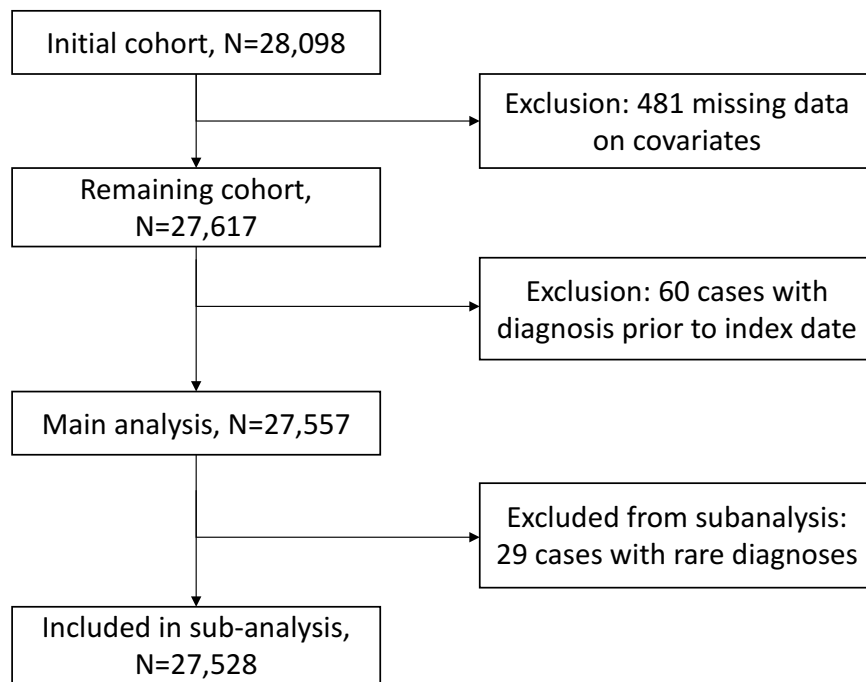

Supplement: S1 Fig — Flow-chart on the total number of cases enrolled on the study and the number of, and reasons for, excluding enrolled cases from final analyses. (PDF) [file pone.0202651.s001.pdf]
