# Supplementary material for: Body composition measurements and risk of hematological malignancies: A population-based cohort study during 20 years of follow-up
Source: PLoS One. 2018 Aug 23;13(8):e0202651. doi: 10.1371/journal.pone.0202651 (PMC6107196; doi:10.1371/journal.pone.0202651)
Supplement: S1 Table — (DOCX) [file pone.0202651.s002.docx]

|  | **n** |
| --- | --- |
| **Myeloid malignancy** | 143 |
| **Acute promyelocytic leukemia** | 1 |
| **Acute myeloid leukemia** | 74 |
| **Myelodysplastic syndromes** | 45 |
| **Chronic myeloid leukemia** | 17 |
| **Myeloid leukemia, unspecified** | 6 |
| **Lymphatic leukemia** | 11 |
| **Acute lymphatic leukemia** | 8 |
| **Lymphatic leukemia, unspecified** | 3 |
| **Multiple Myeloma** | 107 |
| **Lymphoma** | 314 |
| **Hodgkin lymphoma** | 13 |
| **Non-Hodgkin lymphoma** | 301 |
| **Diffuse large B-cell lymphoma** | 46 |
| **Follicular lymphoma** | 44 |
| **Chronic lymphatic leukemia** | 58 |
| **Remaining NHL B-cell lymphoma** | 152 |
| **T-cell lymphoma** | 1 |

**Supplementary Table 1.** Total number of cases with hematological malignancy and the number per subgroup of hematological malignancy. Some cases (n=11) were diagnosed with more than one type of malignancy.
