# Supplementary material for: Body composition measurements and risk of hematological malignancies: A population-based cohort study during 20 years of follow-up
Source: PLoS One. 2018 Aug 23;13(8):e0202651. doi: 10.1371/journal.pone.0202651 (PMC6107196; doi:10.1371/journal.pone.0202651)
Supplement: S2 Table — (DOCX) [file pone.0202651.s003.docx]

|  | **ICD-8** | **ICD-9** | **ICD-10** |
| --- | --- | --- | --- |
| **Myeloid malignancy** | 205 | 205 | C92 |
| **Acute promyelocytic leukemia** | N/A | N/A | C92.4 |
| **Acute myeloid leukemia** | 205.00 | 205.0 | C92.0 |
| **Myelodysplastic syndromes** | N/A | 238.7 | D46.6, D46.7, D46.9, C93.1 |
| **Chronic myeloid leukemia** | 205.15 | 205.1 | C92.1- C92.2 |
| **Myeloid Leukemia, unspecified** | 205.90 | 205X | C92.9 |
| **Lymphatic leukemia** | 204 | 204 | C91 |
| **Acute lymphatic leukemia** | 204.00 | 204.00 | C91.0 |
| **Lymphatic leukemia, unspecified** | 204.90 | 204.90 | C91.9 |
| **Multiple Myeloma** | 203 | 203 | C90.0 |
| **Lymphoma** | N/A | N/A | N/A |
| **Hodgkin lymphoma** | 201 | 201 | C81 |
| **Non-Hodgkin lymphoma** | 200, 202, 204.15 | 200, 202, 204.15 | C82-C85, C88, C91.1 |

**Supplementary Table 2**. ICD codes used to define outcomes.
