# Supplementary material for: Body composition measurements and risk of hematological malignancies: A population-based cohort study during 20 years of follow-up
Source: PLoS One. 2018 Aug 23;13(8):e0202651. doi: 10.1371/journal.pone.0202651 (PMC6107196; doi:10.1371/journal.pone.0202651)
Supplement: S3 Table — (a) Hazard ratios for development of any lymphoid malignancy in 27,557 Swedish men and women followed for a median time of 19.8 years. (b) Hazard ratios for development of any myeloid malignancy in 27,557 Swedish men and women followed for a median time of 19.8 years. (DOCX) [file pone.0202651.s004.docx]

**Supplementary Table 3a**

|  | **HR** | **95%CI** | **HR, p-value** | **LR-test, p-value** |
| --- | --- | --- | --- | --- |
| **Null** |  |  |  |  |
| **BMI** | 1.02 | 0.91-1.15 | 0.72 | 0.72 |
| **WC** | 1.12 | 0.95-1.31 | 0.18 | 0.18 |
| **WHR** | 1.09 | 0.81-1.46 | 0.58 | 0.58 |
| **WHHR** | 0.96 | 0.76-1.22 | 0.76 | 0.76 |
| **WHtR** | 1.06 | 0.92-1.23 | 0.40 | 0.40 |
| **ABSI** | 1.20 | 0.95-1.52 | 0.14 | 0.14 |
| **BFP** | 1.04 | 0.88-1.22 | 0.67 | 0.67 |

**Supplementary Table 3b**

|  | **HR** | **95%CI** | **HR, p-value** | **LR-test, p-value** |
| --- | --- | --- | --- | --- |
| **Null** |  |  |  |  |
| **BMI** | 1.03 | 0.86-1.23 | 0.74 | 0.74 |
| **WC** | 1.12 | 0.88-1.51 | 0.37 | 0.38 |
| **WHR** | 1.13 | 0.72-1.77 | 0.60 | 0.60 |
| **WHHR** | 0.98 | 0.68-1.41 | 0.92 | 0.92 |
| **WHtR** | 1.06 | 0.85-1.32 | 0.60 | 0.60 |
| **ABSI** | 1.22 | 0.85-1.73 | 0.28 | 0.28 |
| **BFP** | 0.96 | 0.74-1.23 | 0.73 | 0.73 |

**Supplementary Table 3.** (a) Hazard ratios for development of any lymphoid malignancy in 27,557 Swedish men and women followed for a median time of 19.8 years. (b) Hazard ratios for development of any myeloid malignancy in 27,557 Swedish men and women followed for a median time of 19.8 years. Estimates adjusted for age and sex. Null model included only age and sex. HRs represent hazard ratios for an one increment increase in standard deviation per anthropometric measure. LR-test for significance of adding respective measure to the null model. Abbreviations: BMI, body mass index. WC, waist circumference. WHR, waist-hip ratio. WHHR, waist-to-hip-to-height ratio. WHtR, Waist-to-height ratio. ABSI, a body shape index. BFP, body fat per cent. HR, hazard ratios. CI, confidence interval. LR-test, likelihood ratio test. Null model included only age and sex.
