# Supplementary material for: Body composition measurements and risk of hematological malignancies: A population-based cohort study during 20 years of follow-up
Source: PLoS One. 2018 Aug 23;13(8):e0202651. doi: 10.1371/journal.pone.0202651 (PMC6107196; doi:10.1371/journal.pone.0202651)
Supplement: S4 Table — (DOCX) [file pone.0202651.s005.docx]

|  | **N** | **HR** | **95%CI** | **P** |
| --- | --- | --- | --- | --- |
| **BMI (kg/m^2^)** | | | | |
| **Continuous** |  | 1.04 | 1.00-1.09 | 0.07 |
| **Categorized** |  |  |  |  |
| **<18.5** | 302 | 1.17 | 0.16-8.52 | 0.16 |
| **18.5-24.9** | 12,693 | (ref) |  |  |
| **25-29.9** | 10,917 | 1.08 | 0.69-1.67 | 0.74 |
| **≥30** | 3,646 | 1.94 | 1.17-3.23 | 0.01 |
| **WHR** | | | | |
| **Above cut-off*** | 15,468 | 1.29 | 0.85-1.96 | 0.23 |
| **WC** | | | | |
| **Continuous** |  | 1.02 | 1.00-1.04 | 0.04 |
| **Above cut-off*** | 11,285 | 1.54 | 1.05-2.25 | 0.03 |

**Supplementary Table 4.** Associations between BMI, WHR and WC and development of multiple myeloma using non-standardized units. Estimates adjusted for age and sex. *Cut-off for WHR defined as >0.8 for women and >0.9 for men, cut-off for WC defined as >80 cm in women and >94 cm in men. Abbreviations: BMI, body mass index. WHR, waist-hip ratio. WC, waist circumference. HR, hazard ratio. CI, confidence interval.
